# SMYD2 Promotes Renal Tubular Cell Apoptosis and Chronic Kidney Disease Following Cisplatin Nephrotoxicity

**DOI:** 10.1096/fj.202402703R

**Published:** 2025-05-20

**Authors:** Siyang Zuo, Huixiong Yuan, Xia Li, Ming Chen, Rui Peng, Siyu Chen, Xue Zou, Yuan Yang, Hehua Long, Zeying Liu, Teng Wang, Bing Guo, Lirong Liu

**Affiliations:** ^1^ Center for Clinical Laboratories The Affiliated Hospital of Guizhou Medical University Guiyang China; ^2^ Guizhou Institute of Precision Medicine Affiliated Hospital of Guizhou Medical University Guiyang China; ^3^ Key Laboratory of Kidney Disease Pathogenesis Research and Transformation Application Guizhou University Guiyang China; ^4^ Guizhou Provincial Key Laboratory of Pathogenesis and Drug Research on Common Chronic Diseases Guizhou Medical University Gui'an New District China

**Keywords:** apoptosis, NF‐κB signal path way, renal tubular epithelial cells, the protein lysine methyltransferase 2

## Abstract

The protein lysine methyltransferase 2 (SMYD2) can affect cell proliferation, differentiation, and survival through methylation of its histone and non‐histone substrates. SMYD2 has been shown to act as an oncogene to promote disease progression in a variety of cancer diseases, but its role in chronic kidney diseases (CKD) pathogenesis has not been fully elucidated. This study aims to investigate the effect of SMYD2 on cisplatin‐induced CKD and its underlying mechanisms. In this study, we found that cisplatin caused severe renal injury in mice, which was accompanied by the up‐regulation of SMYD2 expression. AZ505 treatment significantly down‐regulated cisplatin‐induced renal injury and fibrosis. It also alleviated renal apoptosis and inhibited the phosphorylation level of NF‐κB p65. Conditional knockdown of *Smyd2* achieved similar effects as AZ505. In renal tubular epithelial cells, inhibition or silencing of SMYD2 down‐regulated cisplatin‐induced apoptotic response, while overexpression of SMYD2 induced apoptotic response and activated NF‐κB in response to the up‐regulation of SMYD2 expression. Up‐regulation of SMYD2 induced interaction and phosphorylation of SMYD2 and NF‐κB p65, and inhibition of NF‐κB activation further suppressed cisplatin‐induced NF‐κB activation and apoptosis. The present study suggests that up‐regulation of SMYD2 expression in cisplatin‐induced CKD may promote apoptosis of renal tubular epithelial cells and accelerate the process of renal injury through NF‐κB activation. SMYD2 may serve as a potential target for effective CKD treatment.

## Graphical abstract

SMYD2 mediates the activation of the NF‐κB signaling pathway to promote cisplatin‐induced chronic kidney injury. Cisplatin stimulation induces the upregulation of SMYD2 expression in renal tubular epithelial cells. SMYD2 promotes the phosphorylation of the p65 subunit of NF‐κB through methylation, thereby facilitating the release of inflammatory mediators and inducing cell apoptosis.

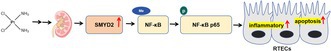

## 1 Introduction

Chronic Kidney Disease (CKD) is a significant public health concern, with its global prevalence and mortality rates increasing annually [1]. Patients with untreated CKD often progress to life‐threatening end‐stage renal failure (ESRD), necessitating kidney transplantation or dialysis, which imposes a psychological and financial burden on patients. Common etiologies of CKD include hyperglycemia, hypertension, and pharmacogenetic renal injury, such as cisplatin therapy [2, 3, 4]. Understanding the pathogenesis of CKD and identifying key molecules mediating its progression are crucial for effective treatment.

Acute kidney injury (AKI) induced by risk factors such as renal ischemia–reperfusion injury [5], cisplatin treatment [6], and sepsis [7], can lead to chronic pathologic changes and progression to CKD. Renal tubular epithelial cell injury and death are common pathologic features of AKI [8]. Cisplatin, a platinum‐based anticancer drug [9], reacts with the DNA of renal tubular epithelial cells to form conjugates that further lead to cell apoptosis and cycle arrest [10], resulting in AKI. Most current studies have focused on AKI resulting from a single high dose of injection cisplatin; however, cisplatin‐based chemotherapy is usually divided into multiple courses or cycles in the clinical management of cancer patients [11]. Persistent renal accumulation of cisplatin induces progression of AKI to CKD [12]. Loss of renal tubular epithelial cells significantly contributes to glomerulosclerosis and tubular atrophy in CKD [13]. There are relatively few reports on the molecular mechanisms associated with renal tubular epithelial cell apoptosis in CKD induced by continuous stimulation with low‐dose cisplatin.

Epigenetics has been shown to be closely linked to human diseases, including CKD. A growing number of studies indicate that epigenetic regulation plays a key role in CKD. Dysregulated expression of pro‐fibrotic factors and inflammatory genes, and altered metabolic status in patients with CKD can induce epigenetic modifications that contribute to the progression of kidney disease [14]. Epigenetic modifications involve changes at the DNA and protein levels without altering the genetic sequence, including DNA methylation, protein post‐translational modifications (PTMs), and non‐coding RNA regulation [15]. The protein lysine methyltransferase 2 is a member of the SET and MYD structural domain (SMYD) family [16], methylates and modifies histone 3 lysine 4 (H3K4) and lysine 36 (H3K36) [17], as well as non‐histone proteins such as nuclear transcription factor κB (NF‐κB), and tumor suppressor p53 [18]. SMYD2 has been reported to act as an oncogene in various cancers such as rectal cancer, lung cancer, and prostate cancer [19]. Our previous study found that inhibition of SMYD2 by targeted drugs may prevent cisplatin‐induced CKD through Smad3 or STAT3‐related signaling pathways [20]. Cui et al. demonstrated that targeting SMYD2 with AZ505 (10 mg/kg) administered after intraperitoneal injection of cisplatin (20 mg/kg) in mice prevented cisplatin‐induced AKI by inhibiting apoptosis and inflammation, as well as promoting cell proliferation. The mice were euthanized 48 h after cisplatin injection [21]. However, the role of SMYD2 in cisplatin‐induced apoptosis of renal tubular epithelial cells in CKD has not yet been studied.

NF‐κB is a pleiotropic nuclear transcription factor that transcriptionally regulates genes related to cellular inflammation, apoptosis, proliferation, and differentiation [22]. In renal diseases, NF‐κB activation regulates oxalate‐induced apoptosis in renal tubular epithelial cells [23], promotes the release of cytosolic inflammatory factors IL‐6, interleukin‐1β (IL‐1β), and TNF‐α [24], and induces the expression of the pro‐fibrotic factors α‐smooth muscle agonist protein (α‐SMA), transforming growth factor β (TGF‐β), and the transcription factor Snail [25], which are known to regulate the disease process. However, studies on SMYD2 regulation of NF‐κB activation in cisplatin‐induced CKD have not yet been reported.

The aims of this study were to assess the protective effect of inhibition or knockdown of *Smyd2* on cisplatin‐induced kidney injury in CKD mice through pharmacological inhibition of AZ505 and conditional knockdown of *Smyd2* in mouse renal tubular epithelial cells. Additionally, we utilized AZ505/BAY11‐7085 intervention, siRNA silencing, and lentiviral overexpression to treat cisplatin‐induced renal tubular epithelial cells in vitro to explore the possible mechanism of action of SMYD2 in cisplatin‐induced CKD.

## 2 Materials and Methods

### 2.1 Main Reagents

DMEM/F12 medium, fetal bovine serum (FBS) were purchased from Invitrogen Gibco, USA; tryptic digest, penicillin–streptomycin mixture were purchased from Biological Industries, Israel; Cis‐platin (CIS), SMYD2 specific inhibitor AZ505, NF‐κB specific inhibitor BAY11‐7085, Streptavidin‐FITC were purchased from APExBIO, USA; Smart‐ECL Enhanced Luminescence Kit was purchased from Changzhou Tiandi Renhe Bio‐technology Co. Ltd.; Hematoxylin and Eosin (HE) kit, MASSON Trichrome Staining Improvement Kit, Glycogen Staining (Periodic Acid Schiff (PAS)) kit, BCA Protein Concentration Measurement Kit, CCK‐8 (Cell Counting Kit‐8, CCK‐8) kit, Streptavidin‐FITC were purchased from APExBIO, USA. high‐efficiency RIPA (Radio Immuno Precipitation Assay, RIPA) lysate, anti‐fluorescence attenuation sealer containing 4′,6‐diamidino‐2‐phenylindole (DAPI), Bromphenol Blue, DL‐Dithiothreitol (DTT), NP‐40 LYSIS BUFFER lysate, and protease inhibitor (Phenylmethanesulfonyl fluoride (PMSF)) were purchased from Beijing Soleilbao Technology Co. TB GreeTM Premix Ex TaqTM II (TLi RNaseH Plus) was purchased from Takara Bio, Japan; Hifair III 1st Strand cDNA Synthesis SuperMix for qPCR (gDNA digester plus) was purchased from Yi Sheng Biotechnology (Shanghai); TUNEL kit (ElabscienceOne‐step TUNEL In Situ Apoptosis Kit) was purchased from Wuhan Elabscience Biotechnology Co. LTD; Goat anti‐Mouse IgG (H + L), CY3 AffiniPure was purchased from Wuhan Pramerica Biotechnology Co. biom; LTL, Biotinylated was purchased from Vector Laboratories Inc. in the USA; Aquaporin 1 (AQP1) (sc‐25 287), Protein A/G PLUS‐Agarose was purchased from SANTA CRUZ biotechnology Co. SANTA CRUZ biotechnology Inc.

### 2.2 Mouse and Animal Models

SPF‐grade C57BL/6 mature male mice were obtained from Zhejiang Vital River Laboratory Animal Technology Co. Ltd. A CKD mouse model was established through weekly intraperitoneal injections of cisplatin (8.5 mg/kg) or an equivalent volume of sterile saline for three consecutive weeks. At the experimental endpoint (week 4), the mice were anesthetized and euthanized, after which urine, serum, and renal tissues were collected for analysis. For pharmacological intervention, mice received AZ505 (a selective SMYD2 inhibitor, 2 mg/kg) through intraperitoneal administration 72 h post‐cisplatin challenge. Parallel control groups were administered vehicle solution (DMSO) using identical injection parameters. For genetic intervention experiments, proximal tubule‐specific SMYD2 knockout mice (*smyd2* ^tecKO^) were generated through crossbreeding SMYD2^*flox/flox*^ (SMYD2^*fl/fl*^) mice with Ggt1‐Cre mice (provided by SAIYE Biotechnology, Suzhou, China), followed by cisplatin‐induced CKD modeling. All animal experiments were performed in accordance with the animal experiment protocols approved by the Animal Protection and Utilization Committee of Guizhou Medical University.

### 2.3 Biochemical Measurement

Renal function parameters including serum creatinine, blood urea nitrogen, and urinary protein levels were quantified using a Cobas 8000 modular clinical chemistry analyzer (Roche Diagnostics) following the manufacturer's standardized protocols.

### 2.4 Histological Analysis

Kidney tissues were fixed with 4% paraformaldehyde (PFA), paraffin‐embedded, and sectioned at 3 μm, and were stained with HE, PAS for histological evaluation; MASSON for evaluating the collagen fiber deposition in the kidney.

### 2.5 Cell Culture and Treatment

Human proximal tubule epithelial cell line‐2 (HK‐2) was purchased from Procell Life Science & Technology Co. Ltd. The cells were cultured with DMEM/F12 medium with 10% FBS and 0.1% penicillin/streptomycin in a 5% CO_2_ humidified incubator at 37°C. The cytotoxicity of AZ505 and BAY11‐7085 was determined by CCK‐8. The cells were treated with various concentrations of AZ505 or BAY11‐7085 for 24 h and then harvested for subsequent analyses. To investigate the roles of SMYD2 and NFκB in regulating HK‐2 cell function, HK‐2 cells were pretreated with AZ505 (40 μM) or BAY11‐7085 (2 μM) for 2 h, followed by treatment with cisplatin (20 μM) for 24 h. The cells were subsequently harvested for further analysis. To elucidate the relationship between SMYD2 and NFκB in HK‐2 cells, the cells were transfected with SMYD2 siRNA (obtained from Shanghai GenePharma Co. Ltd.; sequences are presented in Table 1) or infected with LV5‐SMYD2‐homo lentivirus (obtained from Shanghai GenePharma Co. Ltd), and subsequently pretreated with BAY11‐7085 (2 μM) for 2 h before being treated with cisplatin (20 μM) for 24 h. The cells were then harvested for subsequent analysis.

**TABLE 1:** The sequences of small interfering RNA.

Sequence name	Sense (5′‐3′)	Antisense (5′‐3′)
SMYD2 Homo768	GAGCUGUACAGGAAAUCAATT	UUGAUUUUCCUGUACAGCUCTT
SMYD2 Homo850	CCGGUUAAGAGAUUCUUAUTT	AUAAGAAUCUCUUUAACCGGTT
SMYD2 Homo1342	CGGCAAAGAUCAUCCAUAUTT	AUAUGGAUGAUCUUUGCCGTT
Negative Control	UUCUCCGAACGUGUCACGUTT	ACGUGACACGUUCGGAGAATT

### 2.6 Western Blot Assay

Minced mouse kidney tissue and cultured cells were resuspended in RIPA protein lysis buffer (mixed with PMSF at a 100:1 ratio) on ice. Protein extraction was carried out via ultrasonication, followed by high‐speed centrifugation to isolate the supernatant. Protein concentration was quantified using a BCA assay kit. Samples were mixed with loading buffer, denatured at 100°C for 10 min, and either immediately spotted or stored at −20°C. For Western blot analysis, 20 μg of protein was utilized.

Antibodies used for Western blot analysis included anti‐SMYD2 (21290‐1‐AP), anti‐α‐SMA (14395‐1‐AP), anti‐Collagen I (14695‐1‐AP), anti‐pro‐Caspase 3 (19677‐1‐AP), and anti‐β‐actin (66009‐1‐Ig) were purchased from Proteintech; anti‐Fibronectin (ab2413), anti‐Histone H3 (tri methyl K4) (ab8580), anti‐HistoneH3 (ab1791), and anti‐Bcl 2 (ab32124) were purchased from Abcam; anti‐NF‐κB p65 (D14E12) and anti‐Phospho‐NF‐κB p65 (Ser536) (93H1) were purchased from Cell Signaling Technology; anti‐SMYD2 (sc‐393827) anti‐BAX (sc‐7480) were purchased from Santa Cruz Biotechnology; anti‐Cleaved‐Caspase3 p17 (R23727) was purchased from Zen‐Bioscience; IPKine HRP, Goat Anti‐Mouse/Rabbit IgG HCS were purchased from Abkine; Goat anti‐Mouse/Rabbit IgG (H + L), HRP Conjugated was purchased from Wuhan Pramerica Biotechnology.

### 2.7 Real‐Time Fluorescence Quantitative PCR

Total RNA was extracted using TRIzol Reagent. Subsequently, 1 μg of total RNA was subjected to reverse transcription in a 20 μL reaction system, employing the Hifair III 1st Strand cDNA Synthesis SuperMix for qPCR (gDNA digester plus). RNA expression profiles were analyzed using real‐time PCR on the Applied Biosystems 7500 Fast Real‐Time PCR System in a 10 μL reaction system. The standard procedure for two‐step PCR amplification included the following steps: pre‐denaturation at 95°C for 30 s, followed by 40 cycles of PCR at 95°C for 5 s and 60°C for 34 s, concluding with a melting curve analysis and a cooling step. Each experiment was conducted in triplicate to normalize the mRNA levels of SMYD2 to those of ACTB (primer sequences are presented in Table 2).

**TABLE 2:** The primers used for quantitative real time PCR.

Gene name	Forward (5′‐3′)	Reverse (5′‐3′)
*SMYD2*	TACTGCAATGTGGAGTGTCAGA	ACAGTCTCCGAGGGGATTCCAG
*ACTB*	CATGTACGTTGCTATCCAGGC	CTCCTTAATGTCACGCACGAT

### 2.8 Immunofluorescence Staining

Kidney tissue sections were deparaffinized and subjected to antigen retrieval using pH 9.0 EDTA. HK‐2 cells were fixed with pre‐warmed 4% paraformaldehyde. The paraffin sections and fixed cells were permeabilized with 0.5% Triton X‐100 for 10 min to facilitate denucleation. Subsequently, 5% BSA was utilized to block non‐specific binding at room temperature for one hour. The cells were then stained overnight at 4°C with the following primary antibodies: anti‐SMYD2 (Proteintech, 1:200), anti‐Phospho‐NF‐κB p65 (Ser536) (Cell Signaling Technology, 1:100), anti‐AQP1 (Santa Cruz Biotechnology, 1:100), anti‐BAX (Santa Cruz Biotechnology, 1:50), and anti‐LTL (Vector Laboratories, 1:50). Afterward, a FITC/Cy3 fluorescent secondary antibody was applied at room temperature, protected from light, for one hour. Cell nuclei were stained using DAPI, and imaging was performed using both a ZEISS Axio Imager. A2 light microscope and a ZEISS LSM710 laser confocal imaging system.

### 2.9 TUNEL Staining

The Terminal Deoxynucleotidyl Transferase dUTP Nick End Labeling (TUNEL) assay was employed to detect cell death‐associated DNA fragments characterized by their 3′‐OH DNA ends. Kidney tissue sections were deparaffinized and subsequently washed three times with a 1× proteinase K working solution in accordance with the instructions provided in the Elabscience One‐Step TUNEL In Situ Apoptosis Kit. The proteinase K working solution facilitated cell permeabilization, and nuclei were labeled with TdT Equilibration Buffer. Apoptotic nuclei were then detected using a ZEISS Axio Imager A2 light microscope imaging system.

### 2.10 Protein Immunoprecipitation

Cultured cells were harvested and lysed using NP‐40 cell lysis buffer (containing 0.2 mM PMSF) overnight at 4°C with low‐speed agitation, in accordance with the Protein A/G PLUS‐Agarose kit instructions. The protein supernatants were collected via centrifugation. Anti‐SMYD2 (1 μL) and anti‐NF‐κB p65 (1 μL) antibodies were bound to Protein A/G agar and incubated at low speed overnight at 4°C. After washing the Protein A/G agar with PBS, the protein precipitates were resuspended in supersampling buffer, denatured at 100°C for 10 min, and subsequently analyzed by Western blotting.

### 2.11 Statistical Analysis

Statistical analyses were conducted using GraphPad Prism (version 9.0.2). Data are presented as means ± SD and were subjected to one‐way ANOVA. Multiple means were compared using Tukey's test, and differences between two groups were determined by *t*‐test. For data analysis, a *p*‐value < 0.05 was considered statistically significant.

## 3 Results

### 3.1 SMYD2 Expression Is Upregulated in the Renal Tissues of Mice With Chronic CKD Induced by Cisplatin

**FIGURE 1:**
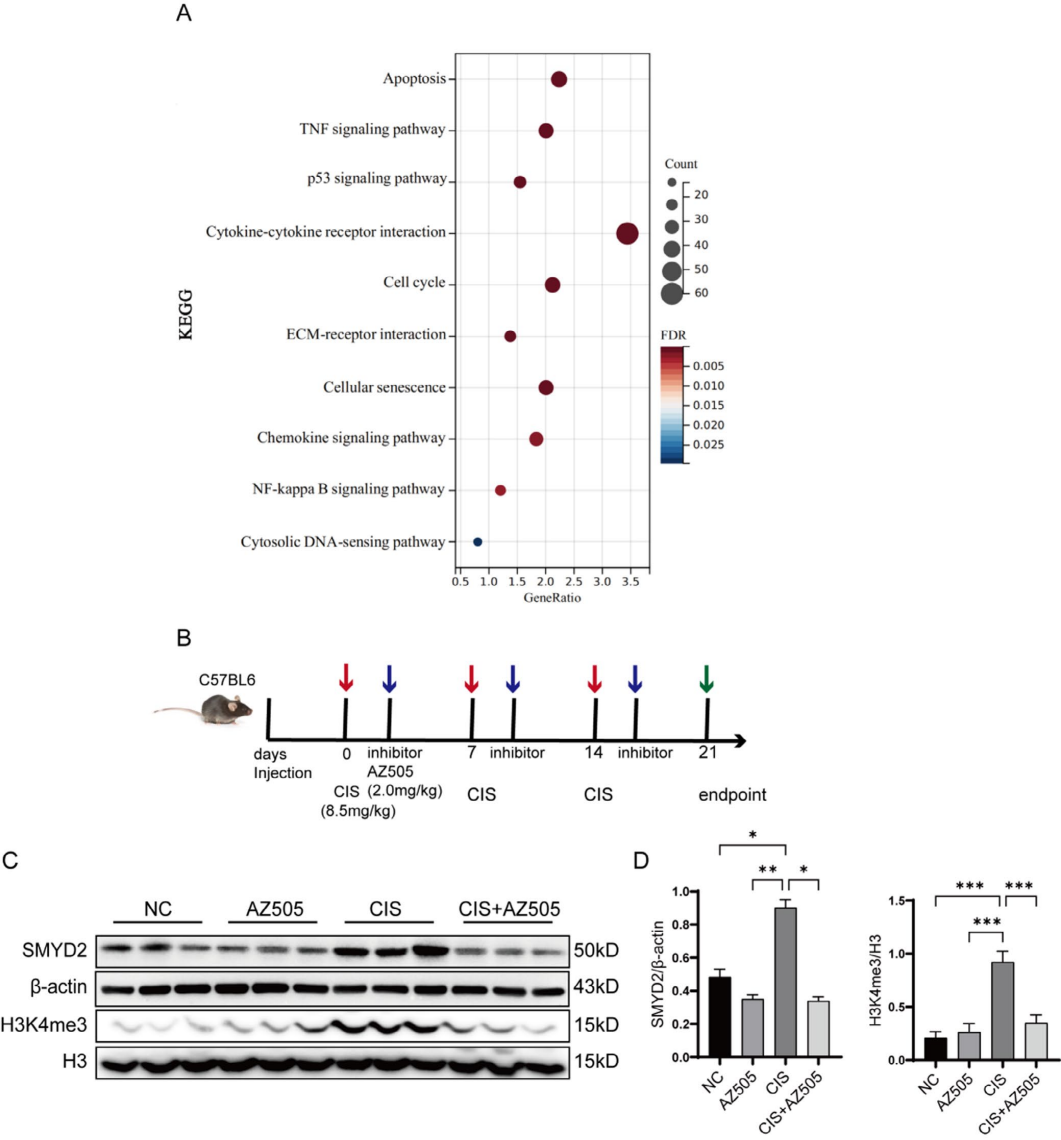
Changes in SMYD2 expression in renal tissues of mice with cisplatin‐induced CKD. (A) Differential expression of genes associated with cisplatin‐induced CKD was analyzed in renal tissues using KEGG enrichment analysis through the Cluster Profiler R package. (B) A CKD mouse model was established via cisplatin treatment in conjunction with AZ505. (C, D) Western blot analysis was conducted to evaluate the expression levels of SMYD2 and H3K4me3 in the kidneys of all groups of mice (*n* = 3). *p < 0.05, **p < 0.01, ***p < 0.001.

The KEGG analysis indicated that gene expression in renal tissues of mice with cisplatin‐induced CKD was predominantly enriched in pathways associated with apoptosis and TNF signaling, among others (Figure 1A). Our previous research demonstrated that SMYD2 plays a role in renal fibrosis in the context of unilateral ureteral obstruction [26], being highly expressed in the kidneys of streptozotocin (STZ)‐induced diabetic mice [27]. Furthermore, the administration of AZ505 mitigated UUO‐induced renal fibrosis and inhibited the proliferative activation of renal mesenchymal fibroblasts, as well as epithelial‐to‐mesenchymal transition (EMT). To elucidate the mechanism of action for SMYD2 in CKD, we established a CKD model in C57BL/6 mice via intraperitoneal injection of cisplatin and subsequently treated the cisplatin‐induced CKD mice with AZ505, a specific SMYD2 inhibitor (Figure 1B). Western blot analysis revealed that the expression levels of SMYD2 and its specific methylation substrate, H3K4me3, were significantly elevated in the kidney tissues of cisplatin‐induced CKD mice, while AZ505 effectively inhibited the upregulation of both SMYD2 and H3K4me3 (Figure 1C,D).

### 3.2 Therapeutic Administration of an SMYD2‐Specific Inhibitor Attenuates Renal Injury in Mice With Cisplatin‐Induced CKD and Suppresses the Upregulation of Extracellular Matrix (ECM) Protein Expression in Kidney Tissues

**FIGURE 2:**
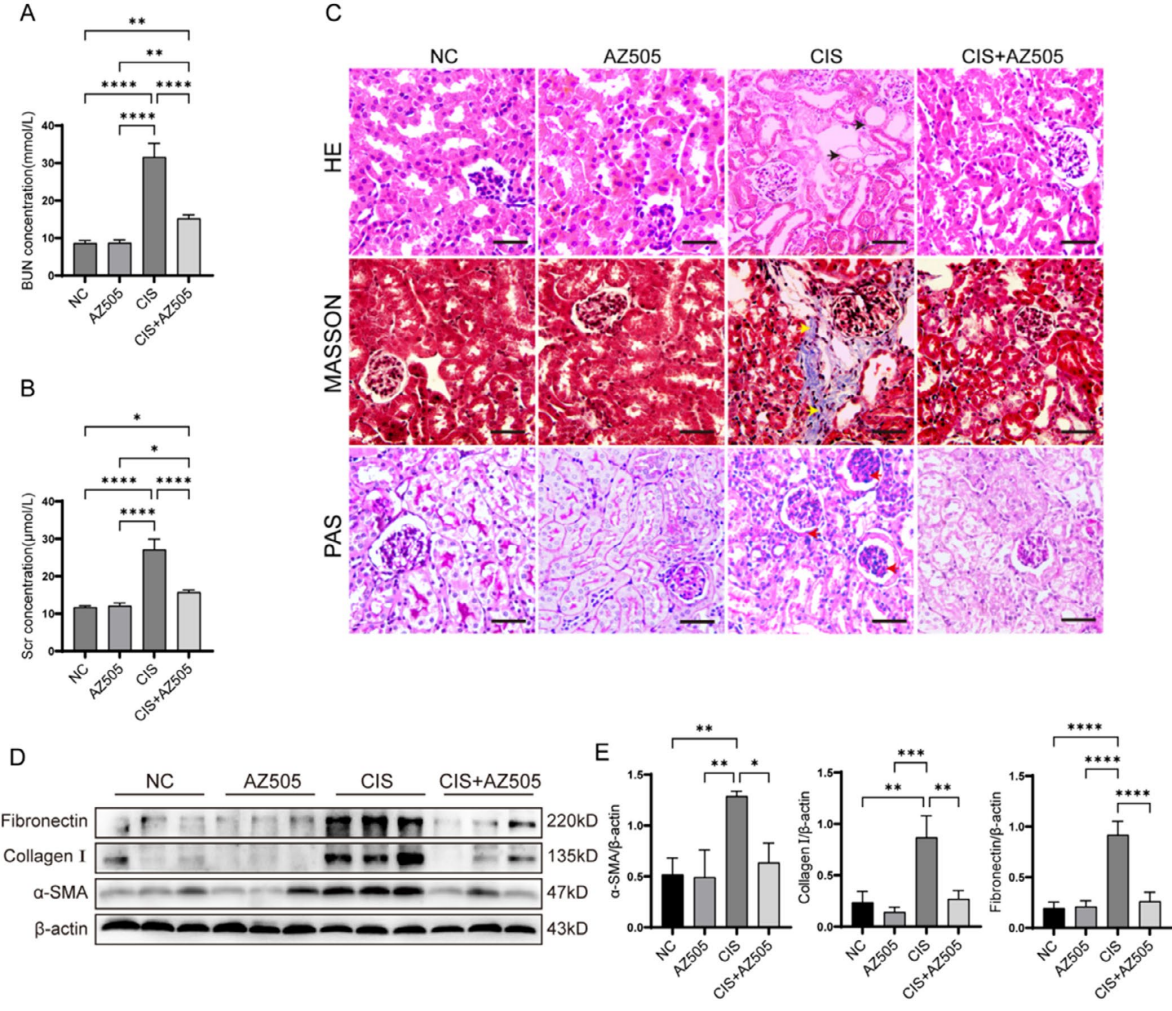
AZ505 mitigates renal injury and suppresses the upregulation of ECM proteins in the renal tissue of mice with cisplatin‐induced CKD. (A, B) Serum blood urea nitrogen (BUN) and serum creatinine (Scr) levels in AZ505‐treated and untreated cisplatin‐induced CKD mice (*n* = 5). (C) Hematoxylin and eosin (H&E), Masson's trichrome, and periodic acid‐Schiff (PAS) staining of renal tissue sections from AZ505‐treated and untreated cisplatin‐induced CKD mice. Scale bar: 10 μm. (D, E) Western blot analysis of ECM protein expression in renal tissues from AZ505‐treated and untreated cisplatin‐induced CKD mice (*n* = 3). *p < 0.05, **p < 0.01, ***p < 0.001, ****p < 0.0001.

We measured blood urea nitrogen (BUN) and serum creatinine (Scr) levels in the serum of mice. The results indicate that cisplatin administration significantly increased serum BUN and Scr levels in mice compared to both the normal control (NC) group and the AZ505 group. Notably, the cisplatin‐induced elevation of BUN and Scr levels was significantly attenuated by AZ505 treatment (Figure 2A,B). Histopathological examination of renal tissues corroborated the biochemical assay findings. Mice in the NC and AZ505 groups exhibited normal glomerular morphology and structure, along with neatly arranged renal tubules. Conversely, cisplatin administration led to renal tubular atrophy, dilation of tubular lumens, collagen fiber deposition, and thickening of glomerular capsule membranes (Figure 2C). AZ505 treatment significantly mitigated the pathological injuries induced by cisplatin, and we observed no significant toxicological effects of AZ505 on renal function and morphology. Furthermore, AZ505 was found to inhibit the cisplatin‐induced upregulation of ECM protein expression as detected by Western blot analysis (Figure 2D,E). These findings indicate that treatment with AZ505 mitigates kidney injury in mice with cisplatin‐induced CKD.

### 3.3 Inhibition of SMYD2 Attenuates Apoptosis in Renal Tubular Epithelial Cells of Cisplatin‐Induced CKD Mice, a Mechanism Likely Mediated by the Suppression of NF‐κB p65 Phosphorylation

**FIGURE 3:**
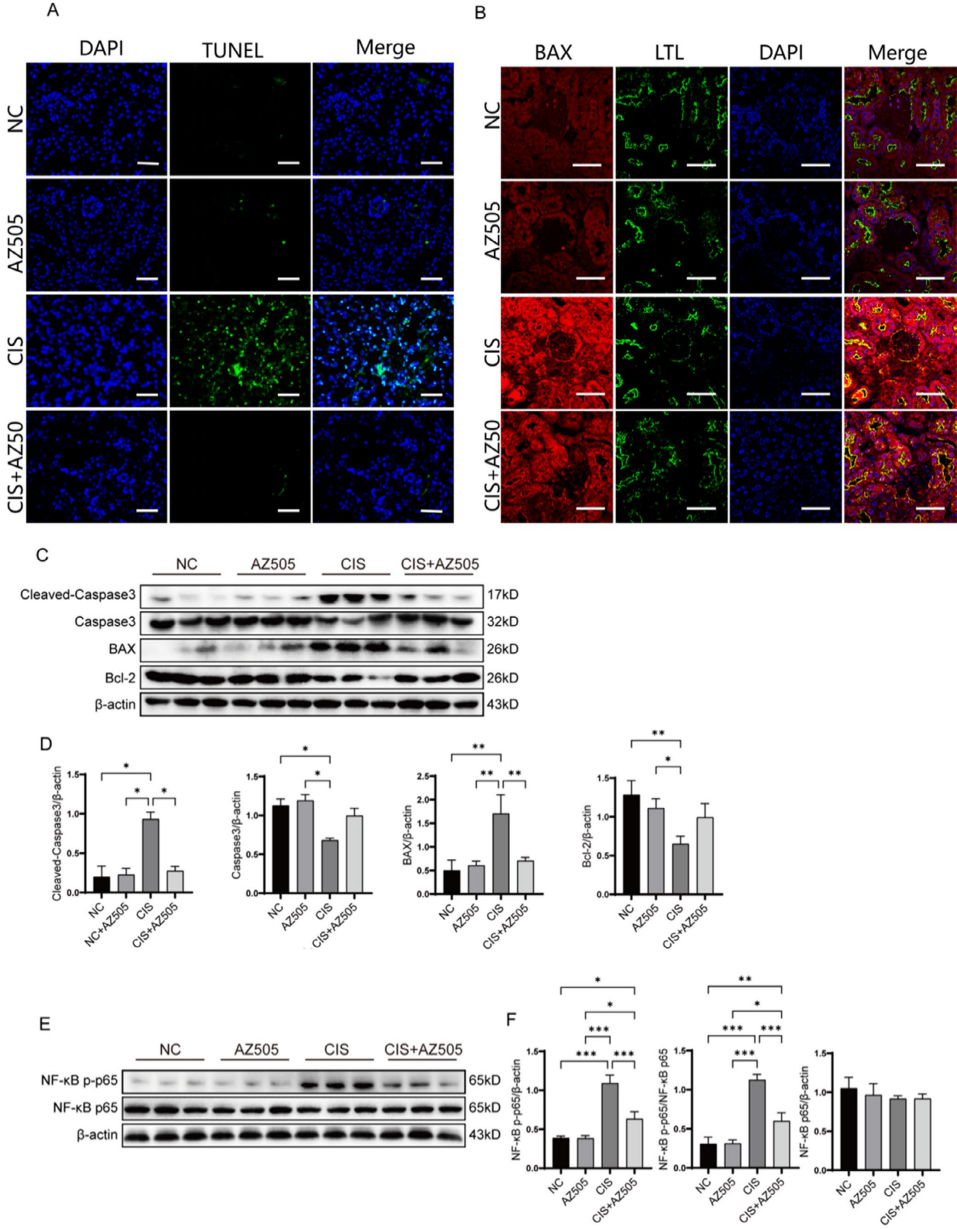
Effects of SMYD2 inhibition on apoptosis and phosphorylated NF‐κB p65 levels in renal tubular epithelial cells from mice with cisplatin‐induced CKD. (A) TUNEL staining of renal tissues from AZ505‐treated and control groups. Scale bar: 50 μm. (B) Immunofluorescence analysis of BAX expression and localization in kidney tissues. Scale bar: 50 μm. (C, D) Western blot analysis of BAX, Bcl‐2, cleaved caspase‐3, and caspase‐3 protein expression in kidney tissues (*n* = 3). (E, F) Western blot analysis of total NF‐κB p65 and phosphorylated NF‐κB p65 (p‐p65) protein levels (*n* = 3). *p < 0.05, **p < 0.01, ***p < 0.001.

Cisplatin accumulation in the kidneys can induce apoptosis in renal tubular epithelial cells [28]. To determine whether AZ505 modulates cisplatin‐induced apoptosis, we performed TUNEL staining, immunofluorescence staining, and Western blot analysis. AZ505 treatment significantly reduced the incidence of TUNEL‐positive cells in renal tubular epithelial cells of CKD mice (Figure 3A) and attenuated BAX fluorescence intensity (Figure 3B). Western blot analysis corroborated these findings: AZ505 suppressed cisplatin‐induced BAX and cleaved caspase‐3 protein expression while upregulating Bcl‐2 and caspase‐3 levels (Figure 3C,D). The results indicate that AZ505 provides protection against apoptosis in renal tubular epithelial cells induced by cisplatin.

To further investigate the regulatory role of SMYD2 in cisplatin‐induced apoptosis in renal tubular epithelial cells of mice with CKD, we analyzed the activity of NF‐κB, a pleiotropic transcription factor that regulates cell growth, differentiation, and apoptosis [29, 30]. Western blot analysis revealed that AZ505 significantly reduced cisplatin‐induced phosphorylation of p65, while total NF‐κB p65 protein levels remained unchanged (Figure 3E,F). These findings suggest that AZ505 attenuates cisplatin‐induced tubular epithelial cell apoptosis in vivo, potentially through suppression of NF‐κB p65 phosphorylation.

### 3.4 Conditional Knockdown of Smyd2 Mitigates Kidney Injury in Cisplatin‐Induced CKD in Mice and Inhibits the Upregulation of ECM Protein Expression

**FIGURE 4:**
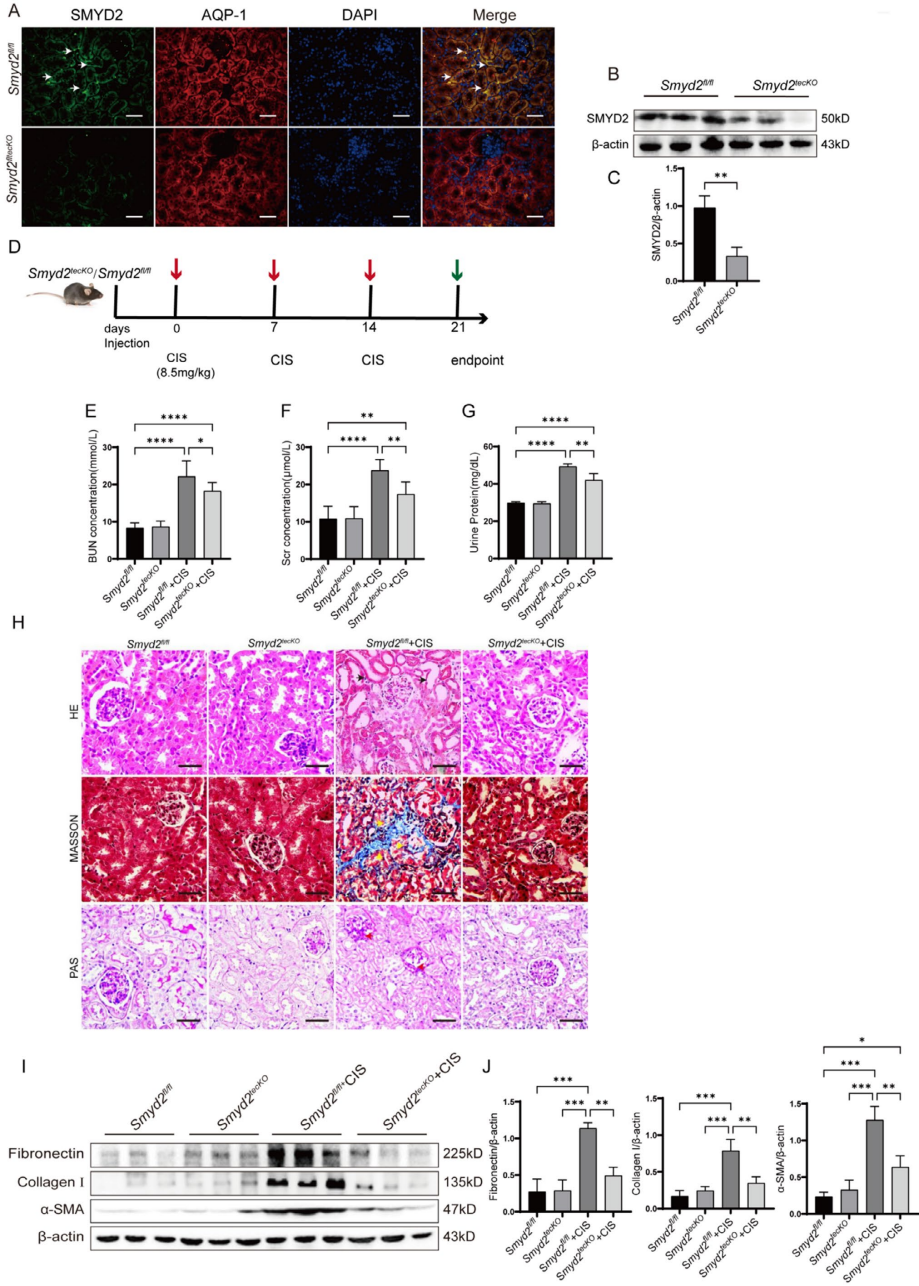
*Smyd2* ^tecKO^ Mitigates kidney injury in cisplatin‐induced CKD mice and suppresses ECM protein upregulation. (A–C) Immunofluorescence and Western blot analysis of SMYD2 expression and localization in renal tissues of *Smyd2* ^tecKO^ mice, with β‐Actin as a loading control (scale bar: 50 μm; *N* = 3). (D) Schematic of cisplatin‐induced CKD model in *Smyd2* ^tecKO^ mice. (E–G) Blood urea nitrogen (BUN), serum creatinine (Scr), and proteinuria levels in cisplatin‐treated *Smyd2* ^tecKO^ mice and *Smyd2* ^fl/fl^ mice (*n* = 5). (H) H&E, Masson, and PAS staining of kidney sections. Scale bar: 10 μm. (I, J) Western blot analysis of renal ECM proteins (α‐SMA, fibronectin, collagen I) in cisplatin‐treated groups (*n* = 3). *p < 0.05, **p < 0.01, ***p < 0.001.

Immunofluorescence staining revealed that SMYD2 is predominantly expressed in renal tubular epithelial cells (Figure 4A). Notably, SMYD2 expression was absent in these cells following epithelial cell‐specific knockdown of the Smyd2 gene within the proximal renal tubule (*Smyd2* ^tecKO^) in mice. Consistent with the immunofluorescence data, Western blot analyses revealed a significant decrease in SMYD2 protein levels in the renal tissues of *Smyd2* ^tecKO^ mice (Figure 4B,C). Given that SMYD2 inhibition ameliorates renal injury in cisplatin‐induced CKD mice, cisplatin was administered intraperitoneally to *Smyd2* ^tecKO^ mice (Figure 4D) to investigate whether cisplatin promotes chronic renal injury by upregulating SMYD2 expression. *Smyd2* ^tecKO^ attenuated cisplatin‐induced chronic renal impairment, as shown by reduced serum creatinine (Scr), blood urea nitrogen (BUN), and proteinuria levels in *Smyd2* ^tecKO^ + CIS mice compared to *Smyd2* ^fl/fl^ + CIS mice (Figure 4E–G), alongside mitigated histopathological damage (Figure 4H). ECM protein levels correlated with morphological improvements: α‐smooth muscle actin (α‐SMA), fibronectin, and collagen I expression were significantly downregulated in *Smyd2* ^tecKO^ + CIS mice (Figure 4I,J). These findings indicate that Smyd2 knockdown in renal tubular epithelial cells alleviates cisplatin‐induced chronic kidney injury, suggesting cisplatin exacerbates renal injury via SMYD2 upregulation in these cells.

### 3.5 *Smyd2* ^tecKO^ May Attenuate Apoptosis in Renal Tubular Epithelial Cells of Cisplatin‐Induced CKD Mice by Inhibiting NF‐κB Signaling Pathway Activation

Given the protective role of SMYD2 inhibition against cisplatin‐induced renal tubular apoptosis in mice, potentially mediated through the inhibition of p65 phosphorylation, we performed a comparative analysis of apoptosis in *Smyd2* ^tecKO^ mice and *Smyd2* ^fl/fl^ mice using TUNEL staining and Western blotting.

**FIGURE 5:**
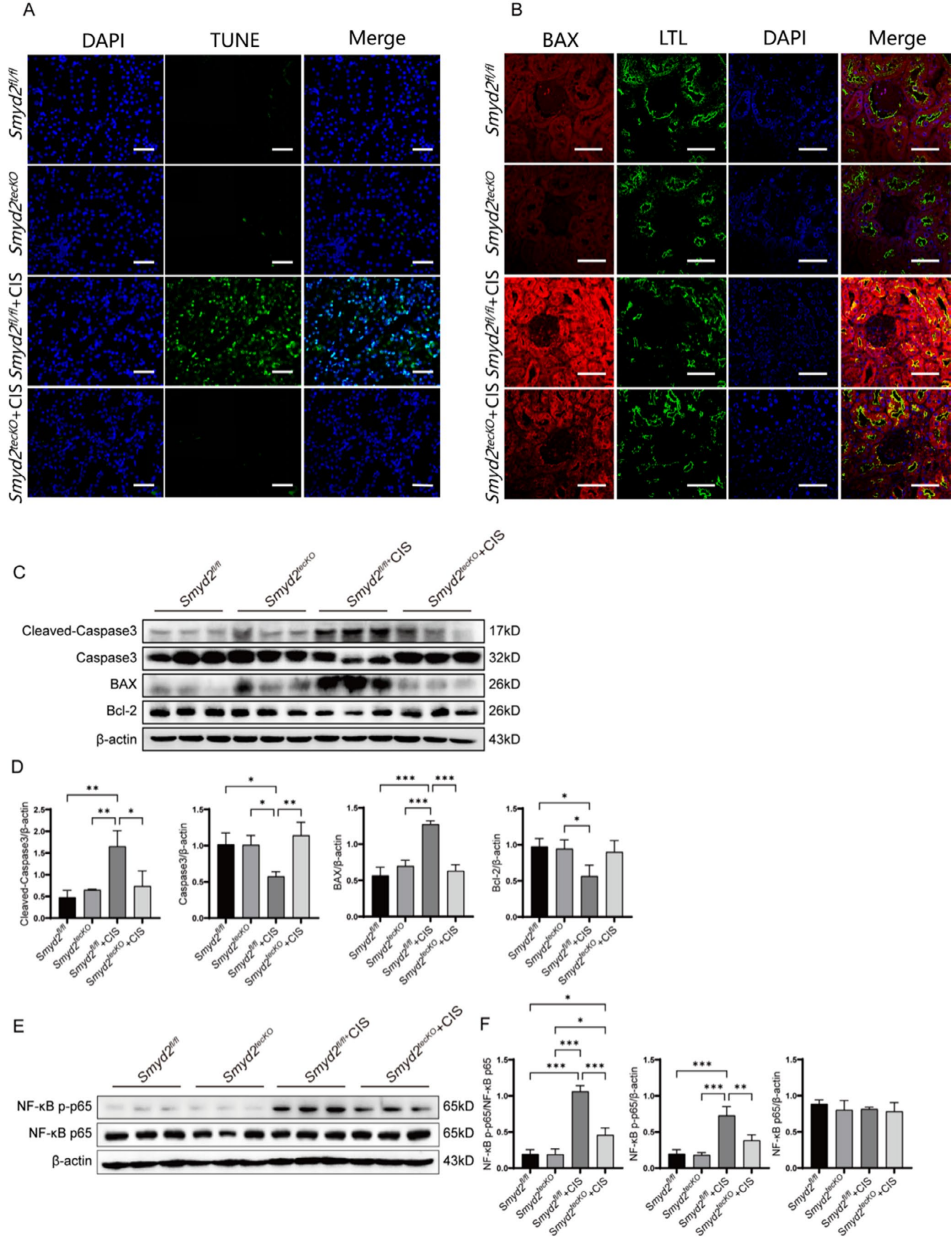
Effects of *Smyd2* ^tecKO^ on apoptosis and phosphorylated p65 levels in renal tubular epithelial cells of cisplatin‐induced CKD mice. (A) TUNEL staining of renal tissues in *Smyd2* ^tecKO^ and *Smyd2* ^fl/fl^ mice. Scale bar: 50 μm. (B) Immunofluorescence analysis of BAX expression and localization in renal tissues. Scale bar: 50 μm. (C, D) Western blotting analysis of renal protein expression levels of BAX, Bcl‐2, cleaved caspase‐3, and caspase‐3 (*n* = 3). (E, F) Western blotting analysis of renal protein expression levels of NF‐κB p65 and phosphorylated p65 (*n* = 3). *p < 0.05, **p < 0.01, ***p < 0.001.

TUNEL staining revealed that *Smyd2* ^tecKO^ reduced cisplatin‐induced apoptosis in renal tubular epithelial cells (Figure 5A). Similarly, *Smyd2* ^tecKO^ decreased the fluorescence intensity of BAX in these cells (Figure 5B). Compared to the *Smyd2* ^fl/fl^ + CIS group, the *Smyd2* ^tecKO^ + CIS group exhibited significantly lower protein levels of BAX and cleaved caspase‐3, alongside upregulated Bcl‐2 and caspase‐3 levels (Figure 5C,D). Furthermore, *Smyd2* ^tecKO^ suppressed cisplatin‐induced phosphorylation of p65 (Figure 5E,F), indicating attenuated NF‐κB activation. These findings indicate that a key mechanism by which *Smyd2* regulates apoptosis in renal tubular epithelial cells of cisplatin‐induced CKD mice involves modulation of NF‐κB signaling.

### 3.6 SMYD2 Inhibition or Silencing Downregulates Apoptosis‐Related Protein Expression via the NF‐κB Signaling Pathway In Vitro

**FIGURE 6:**
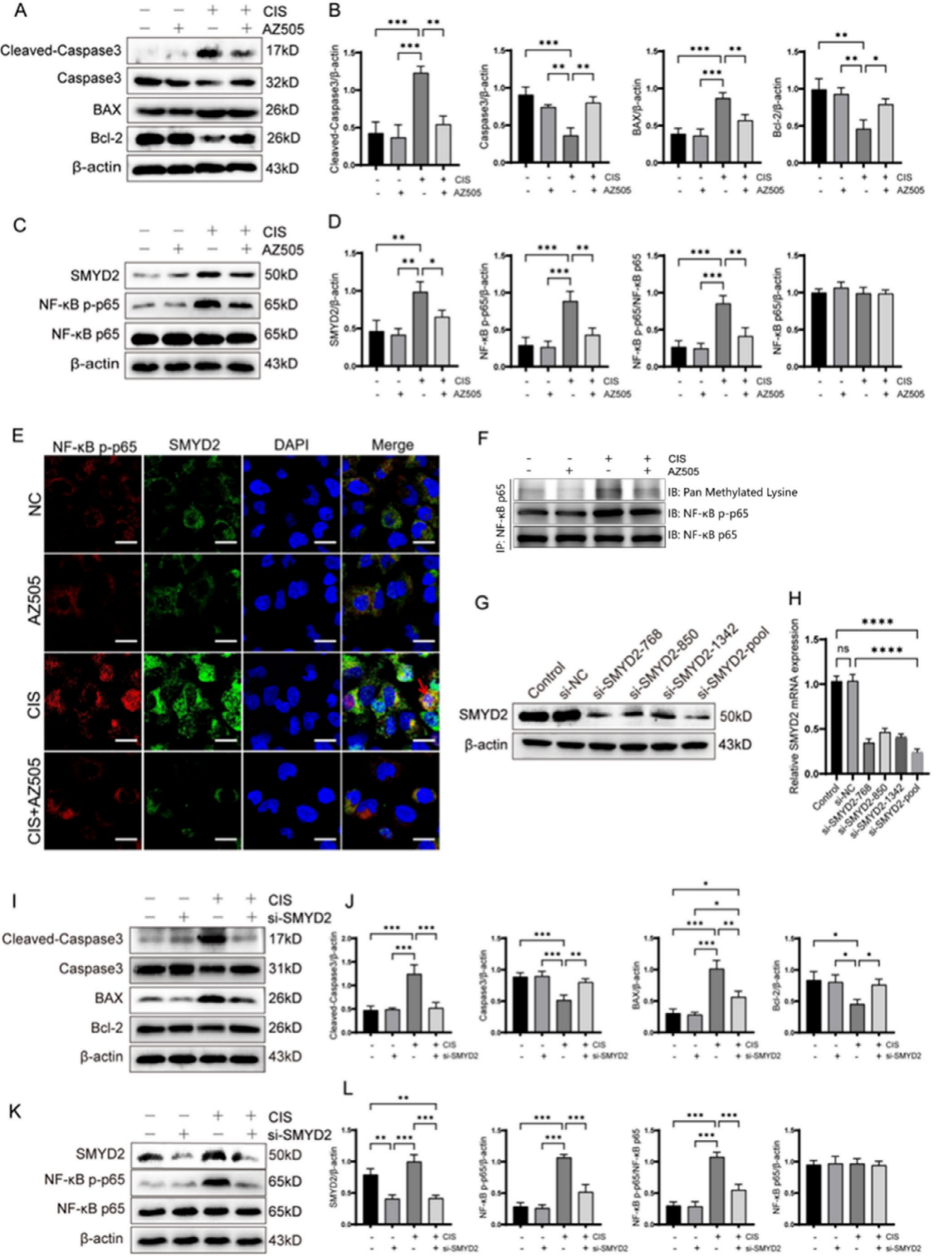
Effects of SMYD2 Inhibition or Silencing on Apoptosis and Phosphorylated p65 Levels in HK‐2 Cells. (A, B) Western blot analysis of BAX, Bcl‐2, Cleaved‐Caspase 3, and Caspase 3 protein expression in cisplatin‐induced HK‐2 cells treated with AZ505 (*n* = 3). (C, D) Western blot analysis of SMYD2, NF‐κB p65, and NF‐κB p‐p65 (*n* = 3). (E) Immunofluorescence detection of SMYD2 and NF‐κB p‐p65 expression and localization. Scale bar: 20 μm. (F) Immunoprecipitation detection of lysine methylation levels in NF‐κB p65. (G, H) Western blot and qPCR analysis of SMYD2 protein and mRNA expression in SMYD2 si‐RNA transfected HK‐2 cells. (I, J) Western blot analysis of BAX, Bcl‐2, Cleaved‐Caspase 3, and Caspase 3 protein expression in SMYD2 si‐RNA transfected HK‐2 cells (*n* = 3). (K, L) Western blot analysis of SMYD2, NF‐κB p65, and NF‐κB p‐p65 expression in SMYD2 si‐RNA transfected HK‐2 cells (*n* = 3). *p < 0.05, **p < 0.01, ***p < 0.001, ****p < 0.0001.

To investigate the regulatory role of SMYD2 in cisplatin‐induced apoptosis in renal tubular epithelial cells, we treated cisplatin‐exposed HK‐2 cells with AZ505, building on prior in vivo findings and hypotheses. Western blot analysis showed that AZ505 (20 μM) significantly downregulated cisplatin‐induced SMYD2 protein levels (Figure 6C,D) and inhibited the upregulation of cleaved caspase‐3 and BAX expression, while restoring Bcl‐2 and caspase‐3 levels (Figure 6A,B). Additionally, AZ505 decreased NF‐κB p‐p65 protein levels and inhibited its nuclear translocation, as confirmed by Western blot and immunofluorescence assays (Figure 6C–E). Immunoprecipitation experiments revealed that cisplatin enhanced lysine methylation of NF‐κB p65, which was attenuated by SMYD2 inhibition via AZ505 (Figure 6F). To further validate these findings, SMYD2 was silenced using siRNA. Transfection with a combination of three distinct SMYD2‐targeting siRNA oligonucleotides achieved optimal knockdown efficiency (Figure 6G,H). Western blot analysis revealed that SMYD2 silencing downregulated apoptotic protein expression (Figure 6I,J) and inhibited NF‐κB p65 phosphorylation (Figure 6K,L) in cisplatin‐treated HK‐2 cells. Collectively, these findings suggest that the inhibition or silencing of SMYD2 mitigates cisplatin‐induced apoptosis in renal tubular epithelial cells in vitro by suppressing the NF‐κB signaling pathway.

### 3.7 SMYD2 Promotes Apoptosis in Renal Tubular Epithelial Cells by Activating the NF‐κB Signaling Pathway

**FIGURE 7:**
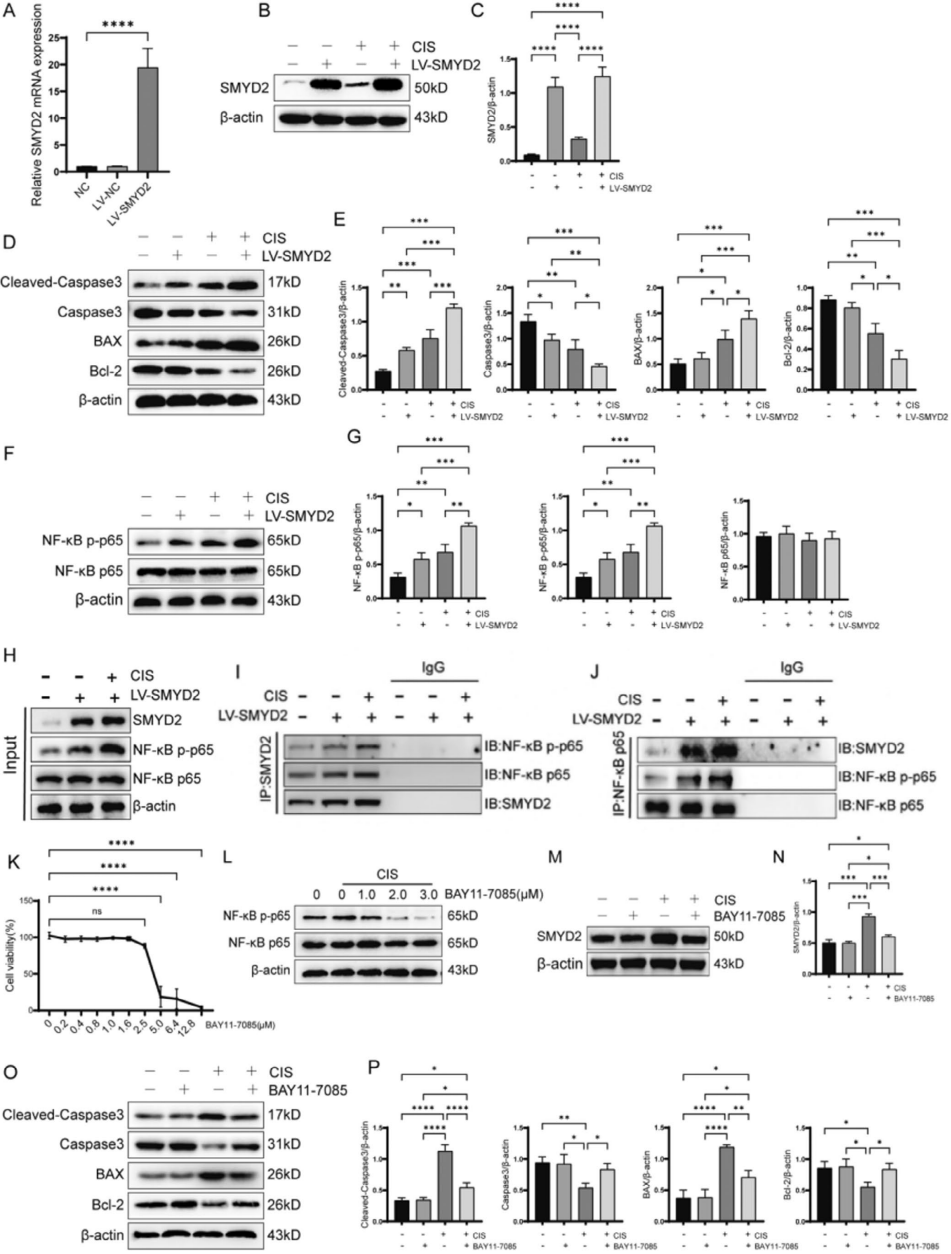
SMYD2 promotes cisplatin‐induced apoptosis in HK‐2 cells via NF‐κB pathway activation. (A–C) Western blot and qRT‐PCR analysis of SMYD2 protein and mRNA levels in LV‐SMYD2‐transfected HK‐2 cells (*n* = 3). (D, E) Western blot analysis of BAX, Bcl‐2, Cleaved‐Caspase3, and Caspase3 (*n* = 3). (F, G) Western blot analysis of NF‐κB p65 and NF‐κB p p65 *n* = 3). (H–J) Co‐IP assay of SMYD2 and NF‐κB p65. (K) CCK‐8 viability assay of HK‐2 cells treated with BAY11‐7085 (*n* = 5). (L) Western blot analysis of p65 phosphorylation in cisplatin‐treated HK‐2 cells following BAY11‐7085 treatment at indicated concentrations. (M, N) Western blot analysis of SMYD2 protein levels in cisplatin‐treated HK‐2 cells after BAY11‐7085 (2 μM) treatment (*n* = 3). (O, P) Western blot analysis of BAX, Bcl‐2, Cleaved‐Caspase3, and Caspase3 protein levels in cisplatin‐treated SMYD2‐overexpressing HK‐2 cells after BAY11‐7085 (2 μM) treatment (*n* = 3). *p < 0.05, **p < 0.01, ***p < 0.001, ****p < 0.0001.

To validate our hypothesis, we generated an SMYD2‐overexpressing HK‐2 cell line via lentiviral transduction and confirmed overexpression efficiency through Western blot and qRT‐PCR analyses. SMYD2 mRNA and protein levels were significantly elevated in transduced cells (Figure 7A–C), with cisplatin treatment further enhancing SMYD2 protein expression (Figure 7B,C). SMYD2 overexpression upregulated Cleaved‐caspase3 and BAX expression (Figure 7D,E), suppressed Bcl‐2 levels (Figure 7D,E), and increased NF‐κB p65 phosphorylation (Figure 7F,G), effects amplified by cisplatin. These findings suggest that SMYD2 overexpression exacerbates cisplatin‐induced apoptosis, likely mediated by NF‐κB pathway activation.

To elucidate SMYD2's role in NF‐κB pathway activation, co‐immunoprecipitation assays revealed SMYD2 interaction with NF‐κB p65 in SMYD2‐overexpressing HK‐2 cells (Figure 7H). Cisplatin treatment strengthened this interaction and augmented p65 phosphorylation.

To assess NF‐κB's functional role in apoptosis, cisplatin‐treated HK‐2 cells were incubated with BAY11‐7085, an NF‐κB inhibitor. BAY11‐7085 (2 μM) exhibited no significant cytotoxicity (Figure 7K) but reduced p65 phosphorylation (Figure 7L). Critically, BAY11‐7085 attenuated SMYD2‐induced upregulation of Cleaved‐caspase3 and BAX (Figure 7O,P), confirming the involvement of NF‐κB in the regulation of apoptosis. Collectively, our findings from both in vivo and in vitro models demonstrate that SMYD2 drives renal tubular epithelial cell apoptosis in cisplatin‐induced CKD by mediating activation of the NF‐κB signaling pathway. 

## 4 Discussion

CKD, which can result from hyperglycemia, hypertension, or the use of nephrotoxic drugs, is characterized by irreversible loss of renal parenchymal cells and renal dysfunction [31]. It has been observed that chronic inflammation, apoptosis, extracellular matrix protein deposition, and reduced renal regenerative capacity are common factors that contribute to the progression of CKD [32]. In recent years, In recent years, there has been increasing interest in studying epigenetic regulation in human diseases. Protein post‐translational modifications (PTMs) are an integral part of epigenetic regulation, and one such PTM is protein lysine methylation, which is mediated by protein lysine methyltransferases [31]. These enzymes can methylate and modify both histone and non‐histone substrates, thereby influencing a wide array of cellular processes and disease progression [32]. For instance, SMYD2 specifically methylates lysine residues on histones H3K4 and H3K36, and regulates various target genes by either inhibiting or promoting their transcriptional activity [17, 33]. Additionally, SMYD2 can affect the proliferation and activation of cystic renal epithelial cells by methylating STAT3 and the NF‐κB p65 subunit [18].

In this study, the application of AZ505 or conditional knockdown of *Smyd2* in renal tubular epithelial cells ameliorated cisplatin‐induced renal injury and alleviated renal tubular epithelial cell apoptosis in cisplatin‐induced CKD mice, which may be related to the inhibition of the NF‐κB signaling pathway. We explored the possible mechanisms by which SMYD2 regulates cisplatin‐induced apoptosis in renal tubular epithelial cells and verified that SMYD2 and its non‐histone substrate, NF‐κB p65, have an interaction, suggesting that SMYD2 may promote the process of CKD by activating NF‐κB‐mediated apoptosis.

SMYD2 has been implicated in polycystic kidney fibrosis [34] and STZ‐induced diabetic mice [26], where the kidneys exhibit high expression levels. The application of AZ505 alleviates polycystic kidney fibrosis and inhibits the proliferative activation of renal mesenchymal fibroblasts as well as epithelial mesenchymal transition (EMT), suggesting that SMYD2 may be a potential therapeutic target for CKD. In this study, we demonstrated that SMYD2 is predominantly expressed in renal tubular epithelial cells, and its protein levels were significantly upregulated following cisplatin treatment. Cisplatin‐induced decline in renal function, renal morphologic‐pathologic injury, and renal tubular epithelial cell apoptosis were alleviated by AZ505. Consistent with the action of AZ505, conditional knockdown of *Smyd2* also significantly ameliorated cisplatin‐induced chronic kidney injury, indicating a critical role for SMYD2 in cisplatin‐induced nephrotoxic CKD. However, the regulatory role of SMYD2 on cisplatin‐induced apoptosis of renal tubular epithelial cells and underlying mechanisms requires further exploration. In vitro experiments on HK‐2 cells showed that AZ505 pretreatment or siRNA silencing of SMYD2 significantly reduced cisplatin‐induced apoptosis, whereas SMYD2 overexpression promoted apoptosis, which was further exacerbated by cisplatin stimulation. This suggests that upregulation of SMYD2 induces apoptosis and promotes kidney injury.

NF‐κB, a nuclear transcription factor regulating various immune and inflammation‐related genes, plays a crucial role in cell development, growth, and apoptosis [35]. The NF‐κB family includes five members: RelA (p65), RelB, c‐Rel, p105, and p100, all sharing the N‐terminal Rel homology domain (RHD). The p65/p50 dimer, generated from p105, is the most abundant and characteristic member of this family, regulating most NF‐κB target genes [36]. Under resting conditions, the p65/p50 dimer forms an inactive trimer with IκB in the cytoplasm. Activation of the NF‐κB pathway leads to the release and nuclear translocation of the p65/p50 dimer, inducing the transcription of target genes [37]. When the NF‐κB pathway is activated, the p65/p50 dimer is detached from p50‐p65‐IκB, and free NF‐κB is further activated through post‐translational modifications of the protein, such as phosphorylation. SMYD2 overexpression promotes NF‐κB pathway activation in inflammatory diseases [38]. Our study found that AZ505 inhibited cisplatin‐induced up‐regulation of the phosphorylation level of NF‐κB p65 subunit in mouse kidney tissues and HK‐2 cells, consistent with its inhibition of cisplatin‐induced apoptotic response. Additionally, we observed in HK‐2 cells that NF‐κB p‐p65 translocated into the nucleus following cisplatin stimulation, and SMYD2 expression was increased in both cytoplasmic and nuclear compartments, and both of them co‐localized in the nucleus and cytoplasm. AZ505 inhibited nuclear expression of NF‐κB p‐p65 and restored its expression in the cytoplasm, also reducing SMYD2 expression. We further detected in *Smyd2* conditional knockout mouse kidneys and SMYD2 silenced or overexpressed HK‐2 cells that the NF‐κB pathway was suppressed with downregulation of SMYD2 expression, and SMYD2 overexpression induced the activation of the NF‐κB pathway. Co‐IP experiments revealed that there was no significant difference in NF‐κB p65 expression among the groups of cells in the Input group. However, the levels of NF‐κB p65 and p‐p65 were elevated in SMYD2‐overexpressing HK‐2 cells within the IP SMYD2 group, suggesting that SMYD2 upregulation promotes the interaction and phosphorylation of NF‐κB p65, further enhanced by cisplatin stimulation. This indicates that elevated SMYD2 expression in cisplatin‐induced CKD may promote apoptosis in renal tubular epithelial cells through activation of the NF‐κB signaling pathway.

NF‐κB signaling pathway activation can mediate AKI or CKD progression through different upstream regulators. For instance, NF‐κB phosphorylation in renal tissues of mice with diabetic nephropathy and high glucose‐treated renal tubular epithelial cells promoted the release of IL‐1β and IL‐18, activating macrophages in the renal mesenchyme and aggravating tubular injury [39]. In the context of calcium oxalate kidney stones, the NF‐κB signaling pathway mediates the induction of autophagy, apoptosis, and mitochondrial dysfunction in renal tubular epithelial cells [24]. In this study, the inhibition of the NF‐κB signaling pathway by BAY11‐7085 mitigated apoptosis in HK‐2 cells, even in the context of SMYD2 overexpression, although it did not entirely abolish apoptosis. This finding suggests that the role of SMYD2 in cisplatin‐induced CKD may be implicated in additional signaling pathways.

Li et al. demonstrated that SMYD2 promotes NF‐κB p65 phosphorylation through methylation at lysine 310 and partially at lysine 221 [18]. However, it is unclear how methylation of lysine at these positions leads to phosphorylation of NF‐κB p65. Lysine methylation can induce subtle conformational changes in proteins, potentially modulating their ability to recruit interaction partners [40]. In NF‐κB p65, lysine methylation may enhance kinase binding, thereby promoting its phosphorylation. In cisplatin‐induced CKD mouse kidneys and HK‐2 cells, we observed that SMYD2 modulates NF‐κB p65 phosphorylation. Furthermore, SMYD2 was found to directly modulate p65 lysine methylation in HK‐2 cells. These findings suggest that SMYD2 acts as a positive regulator of the NF‐κB signaling pathway in CKD. Interestingly, treatment of HK‐2 cells with AZ505 or BAY11‐7085 resulted in reduced SMYD2 expression. This finding suggests the existence of a positive feedback loop between SMYD2 and NF‐κB, consistent with prior observations reported by Li et al. [18]. The activation of the NF‐κB signaling pathway triggers the release of inflammatory mediators; however, its role in regulating apoptosis remains inconsistent. Inhibition of the NF‐κB signaling pathway promotes apoptosis in human cervical cancer cells and glioblastoma tumor cells [41, 42]. However, in cisplatin‐induced AKI and LPS‐induced acute liver injury, the activation of the NF‐κB signaling pathway facilitated apoptosis [43, 44]. In this study, we found that inhibiting NF‐κB slows the upregulation of the pro‐apoptotic proteins BAX and cleaved caspase‐3, as well as the downregulation of the anti‐apoptotic protein Bcl‐2, thereby mitigating cisplatin‐induced apoptosis in HK‐2 cells. Complementary to these findings, experimental data from CKD mouse models confirmed that the activation of the NF‐κB signaling pathway promotes apoptosis in CKD renal tubular epithelial cells. Our study established that cisplatin‐induced renal tissue injury in CKD is mediated by SMYD2 upregulation, which functions as an epigenetic regulator. However, the precise mechanism by which SMYD2 activates the NF‐κB signaling pathway—whether through methylation or alternative mechanisms—remains undetermined and warrants further investigation.

## 5 Conclusion

**FIGURE 8:**
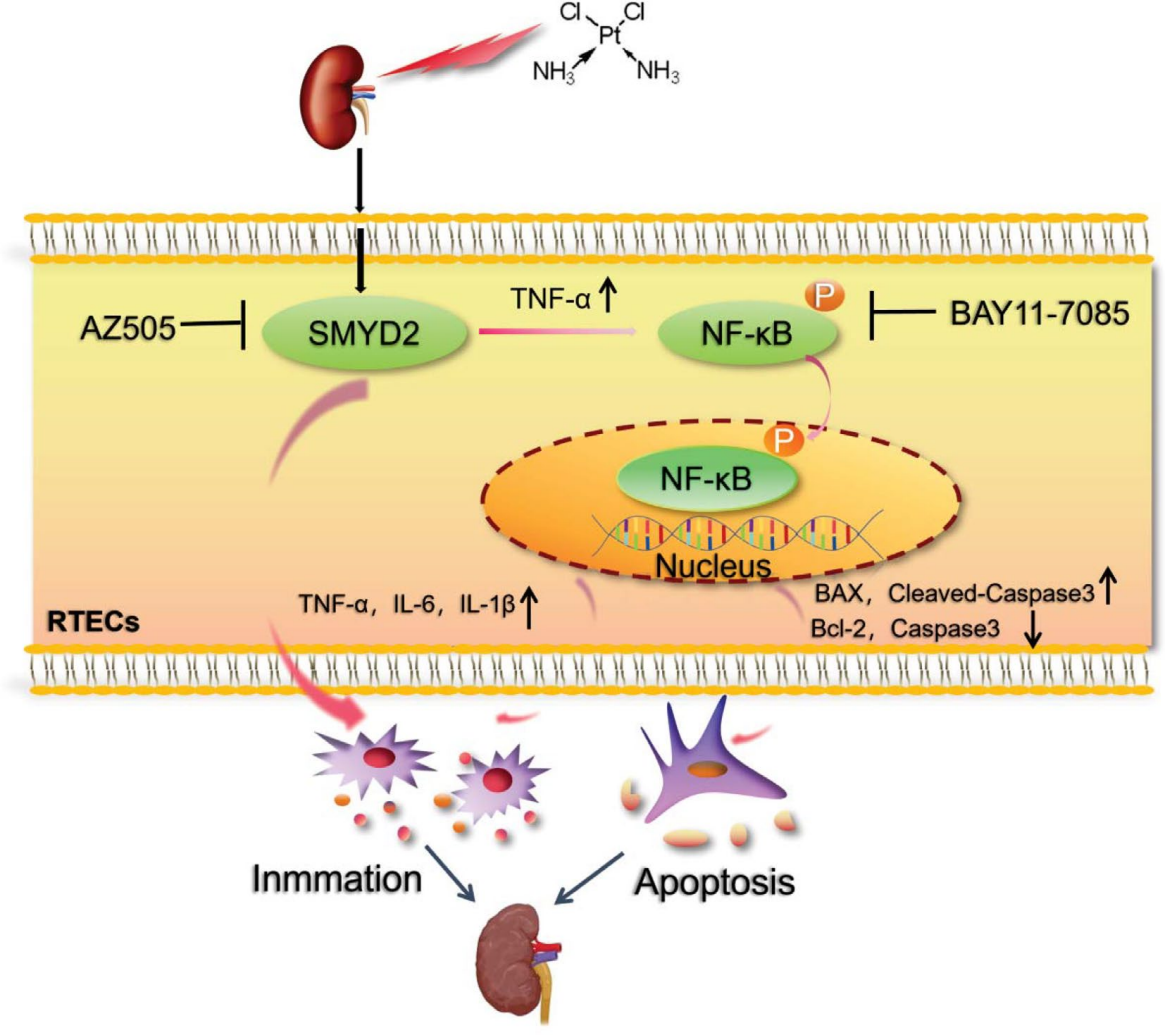
Cisplatin stimulation induces the upregulation of SMYD2 expression in renal tubular epithelial cells, which interacts with NF‐κB and promotes its phosphorylation. This, in turn, enhances the release of inflammatory mediators from the cells and induces apoptosis.

In this study, pharmacological inhibition of SMYD2 with AZ505 or conditional knockdown of *Smyd2* in renal tubular epithelial cells attenuated cisplatin‐induced renal injury by suppressing NF‐κB signaling activation, thereby reducing apoptosis. Mechanistically, cisplatin‐induced upregulation of SMYD2 expression mediated NF‐κB signaling activation, which subsequently exacerbated renal tubular epithelial cell apoptosis (Figure 8). These findings suggest that SMYD2 may represent a potential target for effective clinical interventions in CKD.

## Author Contributions

**Lirong Liu, Bing Guo, and Xia Li:** conceptualization, methodology, project administration; **Siyang Zuo and Huixiong Yuan:** investigation, data curation, writing‐original draft preparation, review, and editing; **Ming Chen, Xue Zou and Rui Peng:** investigation, visualization; **Siyu Chen:** methodology; **Zeying Liu and Yuan Yang:** review, supervision; **Teng Wang and Hehua Long:** software, validation.

## Conflicts of Interest

The authors declare no conflicts of interest.

## Data Availability

All date included in this study are available upon contact with the corresponding author at lirongliu@gmc.edu.cn.
